# An exploratory investigation of psychosocial effects of service dogs on veterans’ families from the perspective of family members

**DOI:** 10.3389/fpsyg.2025.1574445

**Published:** 2025-07-30

**Authors:** Linzi Williamson, Grace Rath, Colleen Dell

**Affiliations:** ^1^Department of Psychology and Health Studies, University of Saskatchewan, Saskatoon, SK, Canada; ^2^Department of Sociology, University of Saskatchewan, Saskatoon, SK, Canada

**Keywords:** veterans, service dogs, family, caregiving, human-animal bond

## Abstract

Research on the psychosocial effects of service dogs (SDogs) on veterans’ family members is relatively limited and often centers veterans’ perspectives rather than those of the family. This exploratory study aimed to examine how Canadian veterans’ family members perceive veterans’ SDog and how they affect different psychosocial outcomes, specifically family quality of life and caregiving. A mixed-methods design utilizing an online questionnaire and follow-up interviews was employed. A non-probability sample of veterans’ family members (i.e., spouses, parents, siblings, friends) were recruited via convenience and snowball sampling methods. Participants (*N* = 35) completed an online questionnaire containing scales measuring their perceptions of and bond with the SDogs, their experience of caregiving, and overall family quality of life. Interviews with veterans’ spouses (*N* = 7) expanded on these topics. We analyzed quantitative data with descriptive and inferential statistics and qualitative data with content analysis. Overall, family members had positive perceptions of and felt bonded to the SDogs. Caregiver scores were relatively high suggesting risk of burnout. Interviewed participants reported no change in their caregiving duties, but they worried less about the veterans because of the SDog. Family quality of life scores were relatively high and SDogs were generally well-integrated into the family, but families seemed to need some support concerning their own emotional well-being. Findings from this study highlight some of the psycho-social benefits of SDogs for veterans’ families from their perspectives. Optimizing these benefits may require awareness of and managing drawbacks related to SDogs, acknowledging limits of the SDog role, and that SDogs’ role can overlap with that of family pets.

## Introduction

Veterans’, or former service members’, posttraumatic stress disorder (PTSD) symptoms can negatively affect family relationships and disrupt family functioning (Dekel and Monson, 2010; Thompson-Hollands et al., 2022). For example, PTSD avoidance symptoms may lead to reduced involvement in family activities, and emotional numbing can inhibit self-disclosure and intimacy (Erbes et al., 2008). Additionally, hyperarousal symptoms, linked to irritability and anger, may lead to aggression and family conflict (Taft et al., 2007a; Taft et al., 2007b). For some, family environments can negatively affect veterans’ PTSD symptoms (Cowlishaw et al., 2014; Ray and Vanstone, 2009; Thompson-Hollands et al., 2022). In other cases, adaptive family environments (e.g., where economic and social decisions are made by consensus; Moen and Wethington, 1992) can reduce PTSD symptom severity (Evans et al., 2009; Evans et al., 2010).

In many cases, veterans’ family members, particularly spouses, adopt caregiver roles to aid with daily tasks and disability management (e.g., reminding veterans to take medication, taking veterans to healthcare appointments, etc.; Bibbo and Proulx, 2019; Cannon and Gray, 2024; National Alliance for Caregiving, 2010; Ramchand et al., 2014; Rattray et al., 2024). Caring for veterans with PTSD can result in family members experiencing caregiver burden or burnout, social isolation, and decreased education and employment pursuits (National Alliance for Caregiving, 2010; Ramchand et al., 2014; Tanielian and Jaycox, 2008). Caregiver burden, for example, can increase the likelihood of spouses experiencing adverse mental and physical health outcomes such as anxiety and depression (Bibbo and Proulx, 2019; Cannon and Gray, 2024; Shepherd-Banigan et al., 2020). However, some spouses have also reported feeling closer to their PTSD-afflicted partner, a sense of pride in caregiving, and individual growth because of their caregiving role and activities (National Alliance for Caregiving, 2010). The concept of caregiver satisfaction, which suggests positive outcomes related to caregiving, such as enjoyment and a sense of purpose, may occur within some veterans’ family (Marks et al., 2002; Sautter et al., 2014).

Increasingly, veterans are relying on service dogs (SDogs) to aid in managing their PTSD symptoms, sometimes as a complement to traditional PTSD treatment models (e.g., therapy, prescription medication; Dell et al., 2022; Leighton et al., 2022; Leighton et al., 2024; Nieforth and Leighton, 2025; Rodriguez et al., 2020a; Williamson et al., 2021a). SDogs are a subtype of animal-assisted human service (AAHS; Moss, 2024) or animal-assisted service (AAS; Binder et al., 2024) defined as “an animal who performs at least one identifiable task or behaviour (not including any form of protection, comfort, or personal defence) to help a person with a disability to mitigate the impacts of that disability, and who is trained to a high standard of behaviour and hygiene appropriate to access public spaces that are prohibited to most animals” (Howell et al., 2022, p. 6). SDogs can provide a holistic model of care and treatment by supporting veterans with their mental, physical, and social health as they are trained to meet the varying health needs of individuals with whom they are paired, including physical, sensory, neurological, and developmental/cognitive impairment (LaFollette et al., 2019; Williamson et al., 2021b; Williamson et al., 2021a). There is a growing line of evidence suggesting that SDogs can decrease veterans’ PTSD symptomology, anxiety, and depression, and improve overall quality of life (Leighton et al., 2022; Leighton et al., 2024; Nieforth et al., 2021; O’Haire and Rodriguez, 2018; Scotland-Coogan, 2019; Vincent et al., 2019; Whitworth and Stewart, 2024; Williamson et al., 2021a).

SDogs are like companion/pet dogs regarding their biophysiological, cognitive, and social needs and potential benefits they can provide their handler. Like companion dogs, SDogs can offer perceived non-judgmental comfort, support, safety, and companionship, and can act as social facilitators to ease socialization (Crowe et al., 2018; Krause-Parello and Morales, 2018; Williamson et al., 2021b). It has been hypothesized that the human-animal bond (HAB), which recognizes the dynamic and influential relationship between people and animals (College of Veterinary Medicine, Purdue University, n.d.), can also promote family resilience (Walsh, 2009).

Like companion dogs, SDogs live in veterans’ homes and commonly share space with veterans’ families. A growing body of literature suggests that this can result in positive and negative psychosocial experiences for veterans’ family (Bibbo et al., 2019; Nieforth et al., 2021; Scotland-Coogan, 2019; Taylor et al., 2013; Williamson et al., 2024). By mitigating veterans’ PTSD symptoms and acting as a relational bridge, SDogs can improve veterans’ ability to interact with people and help them reconnect, improve relationships, and build resiliency within their families (Crowe et al., 2018; Krause-Parello and Morales, 2018; Nieforth et al., 2021; Whitworth et al., 2020; Williamson et al., 2024). Family members may feel bonded to a veteran’s SDog (Bibbo et al., 2019) and experience improved family interactions and relationships due to their familial role (Crowe et al., 2018; Krause-Parello and Morales, 2018; Lessard et al., 2018). They may at the same time be burdened by the work and responsibility of caring for a dog (Krause-Parello and Morales, 2018; Nieforth et al., 2021; Yarborough et al., 2018). The expense and time requirements of SDog training maintenance and care may also be challenging for families (Rodriguez et al., 2020b; Yamamoto and Hart, 2019). Family members, particularly children, may struggle with accepting boundaries when a family member’s SDog is working, including not petting, feeding, or playing with them (Nieforth et al., 2021). In their synthesis of current evidence of AAS/AAHS for military families, Nieforth and Leighton (2025) noted additional factors that can promote and hinder the integration of these interventions, including families having an affinity for animals or previous experience with AAS/AAHS, which could make it easier, and individuals having phobias or allergies to animals, which could make it more difficult.

With the addition of a SDog in the home, the veterans’ family may experience an increase in caregiver burden, or even caregiver satisfaction (Krause-Parello and Morales, 2018; Yarborough et al., 2018). Findings regarding changes in caregiving for veterans’ significant others after the introduction of a SDog have been mixed, with some researchers reporting increased work, responsibility, and stress related to SDog care (Nieforth et al., 2021) and others reporting higher levels of positive emotions, resiliency, and companionship (McCall et al., 2020; Nieforth et al., 2022). For some family members, the benefits of a SDog may outweigh the drawbacks related to their care (Burrows et al., 2016). However, family members can feel left out or jealous of the new relationship between the veteran and SDog and struggle with adjusting to the additional caregiver role (Yarborough et al., 2018).

Much of the research on the psycho-social effects of AAS/AAHS like SDogs on veterans’ families has been investigated from the perspective of the veteran (Neiforth and Leighton, 2024). As such, there is a need to hear directly from veterans’ family to triangulate and increase the validity of research findings. Additionally, this topic has been investigated primarily using samples in the United States (Neiforth and Leighton, 2024) and few published peer-reviewed articles have focused on a Canadian context to date. One Canadian team published a non-peer-reviewed fact sheet highlighting results of interviews completed with a small sample of veterans’ significant others (i.e., spouses/partners, friends; Rath et al., 2022). Significant others reported that the veteran-SDog bond contributed to a stable home environment, improved spousal relationships, reduced stress for themselves and the veterans, and reduced caregiving of the veterans (Rath et al., 2022). Williamson et al. (2024) recently published findings from a mixed-methods investigation of the psychosocial effects of SDogs on veterans’ romantic relationships with their partners from the perspective of the romantic partners. Results indicated there were some improvements to the relationships since the veteran had been supported by a SDog. There were also psychological improvements experienced by the romantic partners, such as less resentment and stress, and increased relaxation, calmness, patience, happiness, and hope for the future. Based on their 2015 research review findings, Veterans Affairs Canada (VAC) concluded that despite the growing body of literature on the importance of families in supporting veterans with service-related conditions like PTSD, there are still knowledge gaps and scant research in Canada on this topic.

Given the relative dearth of research on this topic, particularly in a Canada context and from the perspective of veterans’ family, using a mixed-methods design the current study aimed to explore how Canadian veterans’ family members perceive the impact of veterans’ SDogs on different psycho-social outcomes. A patient-oriented research approach was adopted whereby members of a Research Advisory Committee (RAC), including veterans with SDogs, directed and informed all aspects of the project. Research questions included: What do veterans’ family members feel towards the SDog? In what ways does a veterans’ SDog affect overall family quality of life? Are veterans’ family members experiencing caregiver burden with a SDog in the home?

## Methods

### Research design

We employed a mixed-methods design using an online questionnaire and follow-up interviews.

#### Online questionnaire

Through study advertisements, participants were instructed to access the online questionnaire hosted on Survey Monkey. Participants were invited to complete either the English- or French-version of the questionnaire developed by the first author, in consultation with the study RAC and which was pilot-tested to take approximately 25-min to complete. Participants read about the study process then consented and were directed to the online questionnaire. Upon completing the questionnaire, participants were directed to a debriefing form. Participants were invited to enter a draw for a local community business gift card, and were also invited to complete a follow-up 60-min semi-structured interview with the lead author.

#### Interviews

Interviews ranged in length from 49 to 96 min, with an average of 73 min (*SD* = 18.3). Due to social distancing mandates and because this was a national study, interviews were conducted by telephone and Zoom. Interviews were open to anyone who identified as a family member of a veteran, but only spouses volunteered to participate. Upon interview completion, participants were emailed a debriefing form and offered a local community business gift card.

### Sampling procedures, inclusion and exclusion criteria, and sample size

Participant recruitment was achieved with non-probability convenience and snowball sampling by (1) posting study advertisements on Canadian veteran and SDog organization Facebook groups and (2) emailing study advertisements to Canadian SDog organizations for distribution. Participant inclusion criteria included: (1) English- or French-speaking, (2) 18 years or older, and (3) family member, spouse/partner, or friend of a Canadian veteran paired with a SDog. Online questionnaires were completed by *N* = 35 family members (i.e., spouses, parents, siblings, friends) of veterans and interviews were only completed by *N* = 7 spouses of veterans. Online questionnaire data was collected between February and October 2021 during the COVID-19 pandemic. Interviews took place between July and October 2021.

### Tools used

#### Online questionnaire

Participants provided demographic information (e.g., age, province/territory of residence, relationship to a veteran), background on their experiences with animals (e.g., experience with pets growing up, professional experience with dogs, fear of dogs, dog allergies, whether they had their own SDog), and details about the veterans’ need for a SDog (e.g., PTSD diagnosis, how their SDog was acquired, when their SDog was acquired, SDog breed). Participants completed the Modified Pet Attitude Scale (PAS-M; Munsell et al., 2004), Companion Animal Bonding Scale (CABS; Poresky et al., 1987), Caregiver Burden Inventory (Novak and Guest, 1989), and Beach Centre Family Quality of Life Scale (FQOL; Park et al., 2003). Table 1 describes each of these scales. These scales were selected based on alignment with our research questions (i.e., content validity) and to examine their applicability to the context of SDogs.

**Table 1:** Scale descriptions.

Scale	Description
Modified Pet Attitude Scale (PAS-M; Munsell et al., 2004)	Examines attitudes towards pets using a 7-point response scale (1 = *strongly disagree* to 7 = *strongly agree*) for 18-items across three categories: love and interaction (e.g., I spend time every day playing with my pet), pets in the home (e.g., I like having a pet in my home), and joy of pet ownership (e.g., House pets add happiness to my life). Questions were modified for the current study to inquire about participants’ attitudes toward the veterans’ SDog (e.g., “I frequently talk to my pet” changed to “I frequently talk to my spouse’s service dog”). Six negative-attitude items were reverse-coded and Cronbach’s alpha for the current sample was 0.80, which is considered good (DeVellis, 2003). Each question was scored from 1 to 7 for a total score ranging from 18 to 126, with a higher score indicating more favourable attitudes towards the SDog (Anderson, 2007). Past studies report Cronbach’s alpha scores ranging from 0.76 to 0.90 and mean scores ranging from 89.57 to 102.85 (Min et al., 2019; Phang et al., 2023; Williams et al., 2010).
Companion Animal Bonding Scale (CABS; Poresky et al., 1987)	Examines the human-animal bond using a 5-point response scale (1 = never to 5 = always) across 8 items (e.g., How often do you feel that you have a close relationship with your companion animal?). Questions were modified for the current study to inquire about participants’ bond with the veterans’ SDog (e.g., “How often are you responsible for your companion animal’s care?” modified to “How often are you responsible for the veteran’s service dog’s care”?). Cronbach’s alpha (i.e., internal consistency) for the current sample was 0.83. Total scores result from summed item responses ranging from 8 to 40, with higher scores indicating a greater bond (Poresky et al., 1987). Past studies report mean scores ranging from 24.79 to 30.86 (McGrath, 2014; Nicoll et al., 2008).
Caregiver Burden Inventory (CBI; Novak and Guest, 1989)	A 24-item questionnaire measuring caregiver burden across 5 subscales: (a) Time Dependence; (b) Developmental; (c) Behaviour; (d) Physical Health; (e) Social Relationship; (f) Emotional Burden. Item scores are evaluated using a 5-point scale ranging from 1 (never) to 5 (nearly always). Cronbach’s alpha for the current sample was 0.92. Total scores were calculated by summing the items for a minimum score of 24 and a maximum score of 120, with higher scores indicating a more significant burden and risk of burn-out (Biliunaite et al., 2022). Past studies report Cronbach’s alpha scores ranging from 0.88 to 0.93 and mean scores ranging from 45.22 to 89.79 (Boostaneh et al., 2024; Hosseinnejad et al., 2022; Senturk et al., 2018).
Beach Centre Family Quality of Life Scale (FQoLS; Park et al., 2003)	Examines how people feel about their life together as a family using a 5-point scale (1 = very dissatisfied to 5 = very satisfied) for 21 items across 4 subscales: (1) family interaction (6-items; e.g., My family solves problems together), (2) parenting (6-items; e.g., Family members teach the children how to get along with others), (3) emotional well-being (4-items; e.g., My family has the support we need to relieve stress), and (4) physical/material well-being (5-items; e.g., My family has a way to take care of our expenses). An aggregate total score from the measure using only the 4 subscales ranges from 21 to 105, with higher scores indicating greater family quality of life. Cronbach’s alpha for the current sample was 0.92. The FQOL’s fifth subscale, disability-related support (4-items), was not included in this study as the items pertained to individuals with “special needs” related to school progress and working with a service provider. As such, it was not well-suited for the current sample. Past studies reporting aggregate or mean scores were not located.

#### Interviews

Interview questions can be found in Table 2. Questions were developed with the RAC based on their lived/living experience and expertise. Initially, we did not set out to examine caregiver satisfaction so did not include a scale measuring it. However, given its potential importance to contextualizing our findings we attempted to code for it while analyzing the interviews, specifically looking for examples of variables like enjoyment or sense of purpose (Marks et al., 2002; Sautter et al., 2014).

**Table 2:** Interview questions.

**Family Members’ Relationship with the Veterans’ SDog** If you had any, what were your interactions with the SDog? What activities do you engage in with the SDog? When do you engage in these activities? How often do you engage? How do you feel about the SDog? Do you feel bonded to the SDog? What does your bond look like? **SDog Impact on the Family** What were the biggest challenges & benefits when the SDog first entered your home/life? If there are other animals in the home, how often does the SDog engage with them? How do they engage? Did you incur any financial costs or barriers to having the SDog in your home? If so, what and how much? In what ways has your own or other family members’ mental health been impacted by the SDog? If children are in the home with the SDog, how do the children feel about the SDog? How do they behave with the SDog? What activities do they engage in with the SDog? When do they engage in these activities? How often do they engage? **Family Members’ Relationship with the Veteran** What was your relationship with the veteran like before they got their SDog? How has your relationship with the veteran changed since? Improved? Worsened? If so, in what ways? What role(s) did you play in the life of the Veteran you share your home with/in your family? What did you help them with? Did you engage in caregiving with the veteran before? Are you spending less time caregiving the veteran now? How would you quantify this? What does caregiving look like for you? After the veteran began working with the SDog, how were social gatherings/get togethers? Has there been any change since the introduction of the SDog?

### Data analyses

A third-party translator completed the French translation of the questionnaire and participant responses. Linguistic validation was achieved by the first author via reverse translation using an online neural translation service (www.deepl.com). Quantitative results were analyzed using descriptive statistics (e.g., means, frequencies) and inferential statistics (e.g., ANOVA). Qualitative data was analyzed using content analysis. Inter-coder reliability was achieved with authors one and two independently conducting deductive analyses using Saldaña’s (2013) content analysis coding guide. The authors first independently coded each of the interviews, then met to collaboratively discuss and review their coding, resolve any discrepancies, and finalize the codes.

### Ethical considerations

This study was approved by the authors’ institutional research ethics board (Beh-2471).

### Participants

The average age of online questionnaire participants was 52.5 years (range = 30 to 72). Friends of a veteran (*n* = 2, 5.7%) reported knowing the veteran in their life on average 7.5 years (*SD* = 3.54, range = 5 to 10 years). Most participants grew up with pets (*n* = 21), primarily cats (*n* = 17) and/or dogs (*n* = 18), and most currently had others pets in the home (*n* = 21), mostly cats (*n* = 10) and/or dogs (*n* = 11). Most participants had no professional working experience (e.g., training, boarding, veterinary care) with dogs (*n* = 21) or other animals (*n* = 24). Regarding participants’ other experiences with dogs, two reported being allergic to dogs, 10 were bitten by, and five were afraid of dogs. Half the sample indicated they never had their own SDog (*n* = 18) while the other half (*n* = 17) currently or previously had their own SDog.

Of the spouses (*n* = 27) who completed the questionnaire, 17 (63%) reported raising children with their veteran spouse, and no additional information on children was gathered as this was not a primary focus. Spouses reported being in a relationship with their veteran partner on average 23 years (*SD* = 12.5, range = 2 to 52 years). Questionnaire participants reported the veteran in their life obtained their SDog between 2012 and 2021, with most (*n* = 17; 48.6%) reporting the SDog was acquired within the prior 4 years of the study, primarily for PTSD (74%), followed by anxiety (57%), depression (49%), physical mobility issues (20%), panic disorder (14%), moral injury (11%), physiological health disorder(s; 11%), cognitive impairment (11%), and poor outcome with medication regimen (9%).

The average age of interviewees was 49.4 years (range = 34 to 72). Each interviewed spouse reported being married to their veteran romantic partner (relationship length *M* = 24.5 years). Among interviewed spouses, two had no children, two had older children (i.e., teenagers or in their 40s), and three had younger children (i.e., under the age of 10). Of those with younger children, one participant had three children while the others each had two with their veteran partner. PTSD symptom management was the most common reason interviewed spouses’ reported their veteran partner required a SDog. Additional participant demographics are summarized in Table 3.

**Table 3:** Participant demographic frequencies.

Demographic categories	Questionnaire participants (*N* = 35)	Interviewed participants (*N* = 7)
Significant Other Identity		
Spouse	27	7
Friend	4	0
Mother	2	0
Father	1	0
Sibling	1	0
Sexual Orientation		
Heterosexual	24	7
Lesbian	2	0
Bisexual	1	0
Declined to disclose sexual orientation	8	0
Ethnic Identity		
White	21	7
Native Quebecer	1	0
First Nations	1	0
Christian	1	0
Not indicated	2	0
Province of Residence		
Ontario	12	2
Quebec	4	0
British Columbia	3	1
Manitoba	5	1
Saskatchewan	3	2
Nova Scotia	1	0
Alberta	1	0
New Brunswick	0	1
Not indicated	7	0
Military Membership		
Veteran	20	0
Cadet Organizations Administration and Training Service (COATS)	8	0
SDog Acquisition Mode		
Purchased a partially trained SDog from an organization	4	0
Received a fully trained SDog from an organization by donation	3	2
Received a partially trained SDog from an organization by donation	6	0
Received an untrained dog from a SDog organization by donation that the veteran trained themselves	2	3
Family dog was trained as a SDog by the veteran themselves	6	2
Family dog was trained as a SDog primarily by the veteran, with some support from an SDog organization or professional trainer	9	1
Purchased a dog and sent them to a school to be formally trained as a SDog	2	0

One interviewed spouse’s veteran partner had two SDogs, each acquired differently, which increased the overall count by one for the “SDog Acquisition Mode” variable. At the time of data collection, *n* = 25 questionnaire participants and *n* = 7 interview participants reported living with the veteran in their life. Most veterans (*n* = 22) acquired their SDog within the 6 years prior to their family members’ study participation.

## Results

### Attitudes towards and bond with veterans’ SDog

The overall mean score for the PAS-M was *M* = 108.68 (*SD* = 9.96) and for the CABS the average score was *M* = 20.68 (*SD* = 5.43), suggesting significant others had positive attitudes towards and felt bonded to the veterans’ SDog (Munsell et al., 2004; Poresky et al., 1987). Interview accounts echo these scores. Spouses reported loving the SDogs, being thankful for them, and respecting the work they did. Some, but not all, spouses reported feeling bonded to the SDog and personally experienced benefits from interacting with them, including reduced stress and increased comfort.

“I would say in that beginning phase, when [veteran] wasn't bonding with [SDog]… I felt like I bonded with [SDog] instantaneously because, you know, I wanted to welcome him into the family and [veteran] kind of [gave SDog] the cold shoulder a little bit. And so I immediately wanted to include [SDog] and like I started talking to him right away. And yeah, I would say instantly [I felt bonded to SDog]. I just, I wanted I really wanted it to go.” – Interview Spouse 6

“If she wasn't, not that she wouldn't become certified, but the idea that, ‘Oh, she could not be part of our family’ was devastating. I was just like, ‘no’, that's the type of personality I am. It's like, you've come through our door, you've joined our family and you're one of us now and so she very much is very, very bonded to myself, my children, our family.” – Interviewed Spouse 3

“[SDog] will follow me around the house just like a loving dog.” – Interviewed Spouse 4

However, two spouses indicated they had intentionally attempted to not bond with the dogs because they are the veterans’ SDog.

“I do [feel bonded to SDog to] a certain degree, I do, you know, and like I said, I consciously went into [training SDog] not keeping the arms wide just so that his primary bond would be with [veteran] and that's important.” – Interviewed Spouse 1

“I love [SDog], but he loves [veteran] more.” – Interviewed Spouse 5

### Family quality of life

The aggregate mean score for the FQoLS was *M* = 89.53 (*SD* = 15.06), suggesting family quality of life was relatively high (Park et al., 2003). Repeated measures ANOVA showed a significant difference among the FQoLS subscales, *F*(3,60) = 65.03, *p* < 0.001, with participants scoring the lowest on the Emotional Wellbeing subscale (*M* = 14.2, *SD* = 3.86), followed by the Physical and Materials Wellbeing subscale (*M* = 22.00, *SD* = 3.85), Family Interaction subscale (*M* = 24.76, *SD* = 4.73), and Parenting subscale (*M* = 28.57, *SD* = 6.37), with all subscales significantly differing from one another at *p* < 0.01. Interview questions did not focus on the overall family quality of life, and instead centred the effects of the SDog on the family. Interview accounts suggest that having a SDog in their home was positive for their family. SDogs were described as part of the family, having a special place in the family, being an integral part of the family, positively changing the atmosphere in the home, being well-suited to the home, improving communication among the family, bringing normalcy to the home and family, taking care of the whole family, and being loved by the entire family. Some spouses indicated when the service vest is off at home, and it is time to take a break from working, the SDogs displayed therapy dog-like qualities towards them and their children, such as providing comfort through cuddling. One spouse noted the SDog seemed particularly drawn to people in distress and sought to comfort them, while another spouse reported the SDog is often torn between staying with the veteran or tending to the children, which may be an indication of where the dog is with their SDog training or may speak to the personality of the dog. One spouse noted how the SDog helped their family manage the loss of their previous family dog, and two spouses reported the SDogs helped their children overcome a fear of dogs.

“You can almost forget sometimes that she’s a service dog when her vest is off because she does integrate into our family so well. I definitely think the kids and myself have received positive mental health support by having [SDog] in the household. Especially being a family who has had dogs in the past…You can sit and pet her gently and then she becomes this focus and this tool for all of us that we can kind of just channel our emotional energy to what she’s able to provide.” – Interviewed Spouse 3

Reported drawbacks to having a SDog were managing veterinary bills, food costs, and multiple pets in the home. Two spouses mentioned the only SDog-specific drawback was difficulty affording training program fees (e.g., SDog equipment).

### Caregiving burden

The CBI mean score was 62.13 (*SD* = 19.30, range = 26–98), indicating participants had more significant burden and were at risk of burnout (Biliunaite et al., 2022). However, most interviewed spouses reported their workload decreased and they provided less of a caregiving role overall since their veteran partners started working with a SDog. One spouse reported experiencing a 30–40% decrease in her caregiving duties for her veteran partner, while two spouses noted they had trouble letting go of some of their caregiving management. Everyday tasks spouses completed for the veterans included reminding them to take medication, eat, and sleep, managing their appointments and daily routine, driving them everywhere, maintaining the home (i.e., completing chores), and completing most parenting tasks. Specific tasks some of the SDogs took on for the spouses included supporting veterans during panic attacks, waking them up during nightmares, accompanying them while driving, helping them get out of negative head spaces, providing support in social situations, and keeping them company. However, spouses noted there are some tasks SDogs simply cannot provide support in, including helping manage schedules and appointments. Some spouses reported caring for the SDogs’ needs, including walking, feeding, brushing, playing, training, taking them to veterinarian appointments, and helping the veterans bond with their SDogs, which added to their work load. Spousal accounts during interviews did not center caregiver satisfaction (e.g., enjoyment, sense of purpose; Marks et al., 2002; Sautter et al., 2014) related to the veterans or SDogs beyond indicating they had positive feelings towards the SDog as noted above.

Beyond decreasing aspects of caregiving for some participants, each spouse reported the SDogs decreased their mental load. Specifically, they worried less, felt less stress in their bodies, could relax more, could “shut their brain off,” hovered over the veterans less, felt less concerned about the veterans’ safety, felt less pressured, felt less resentment towards the veteran, felt more confident in the veteran’s ability to manage themself, and found they made fewer excuses for the veteran’s behaviour. Although, two spouses reported carrying the traumas their veteran partners shared with them, and some felt they had developed vicarious trauma or PTSD.

“I think [the SDog] helps. It certainly helped my stress level. I feel much more relaxed, even though he [SDog] is another living thing in the home that I have to care for. I know he’s not my full responsibility. It makes me feel good to have them here.” – Interviewed Spouse 7

“I just don’t have to worry” [decreased cognitive load]; “now I can actually go to work, focus on work. So, it’s just taking that pressure and fear off me that something bad will happen. It’s not there anymore or even close to what it was.” – Interviewed Spouse 2

“That’s been an amazing piece for me to shut off my brain and be like, is he okay? Is he okay? Where are we at? And it’s like, she [SDog] will let us know. Even if she doesn’t have the vest on, she’s always aware of what’s going on with him. And that, that has been great for me” – Interviewed Spouse 3

“Mostly I would say the psychological stuff is the part of caregiving [for the veteran] that I feel is the heaviest load.” – Interviewed Spouse 6

## Discussion

This study explored the perceived impact of veterans’ SDog on family quality of life and caregiving responsibilities from the perspective of veterans’ family members (e.g., spouses, family members, friends). The goal was to determine if veterans’ family felt bonded to and/or burdened by the SDog and how the SDog affects family quality of life.

Evidence from the PAS-M, CABS, and interview content suggests the SDogs were integral family members who were loved by and provided benefits for the entire family. Past reports on PAS-M mean scores have ranged from 90 to 102.85 (Creary, 2017; Kim et al., 2020; Leos et al., 2023; Phang et al., 2023), with our study mean score falling slightly above this range, and CABS mean scores have ranged from 25.04 to 30.86 (McGrath, 2014; Nicoll et al., 2008), with our study mean score falling below this range. Many SDogs seemed to play dual roles in the current study – SDog for the veterans and companion/pet, or sometimes therapy, dog for family members. Studies have reported numerous physical, psychological, and social benefits of companion/pet dogs (Hart, 2010; Hussein et al., 2021; Lass-Hennemann et al., 2022; Maharaj and Haney, 2015; Yamamoto and Hart, 2019) and SDogs for individuals and their families (Crowe et al., 2018; Krause-Parello and Morales, 2018; Nieforth et al., 2021; Rath et al., 2022; Whitworth et al., 2020). SDogs are often categorized as being trained to help one individual with their disability and remain focused on their handler and attuned to their needs (Audrestch et al., 2015). With the current study, the dual role of the SDogs (i.e., service and companion) may be due to the SDog and veterans needing further training to ensure role specificity. SDog training usually takes about 2 years with continuous skill maintenance afterwards (Canadian Association of Professional Dog Trainers, 2021). A period of adjustment to owning and training a SDog in comparison to a companion dog has also been reported for families (Hellings et al., 2022; Nieforth et al., 2021). The dual role of the SDogs may also reflect the realities of having a dog integrated into a family home and not necessarily a lack of training as well as half the sample being experienced SDog handlers and there being multiple SDogs in some homes. Depending on the veterans’ disability-related needs, some SDogs may have more downtime to focus on other activities. Some dogs are also naturally more social and can form close relationships and bond with multiple humans (Marcato et al., 2022). SDogs have been reported as highly sensitive to human attentional states, emotional expressions, and cues (Call et al., 2003; Kaminski et al., 2009; Kujala, 2017) and facilitators of social interaction, development of social-cognitive skills, and active participation in the home and community (Catala et al., 2018; Herlache-Pretzer et al., 2017; Rath et al., 2022; Valentine et al., 1993; Williamson et al., 2021b). However, it is possible SDogs may be unable to address the disability needs of their handler if they are distracted by other individuals living in the home. In these cases, this might suggest the dogs are not well-suited to the handler or more training and home-integration is required. There may be animal welfare benefits (e.g., freedom to express normal behaviour; Mellor, 2016), to redefining the standardized role of a SDog (i.e., task-trained to assist a handler with a disability) to account for the realities of living in a familial home, the social nature of dogs, and their capacity to bond with humans. There may be utility, for example, in standardizing the inclusion of handlers’ families in all SDog training programs and providing education on the HAB which is not currently a common practice in Canada (Williamson et al., 2025).

Results for the FQoLS suggest that family quality of life was good overall, but families could use some support concerning emotional well-being. Previous studies have reported FQoLS mean scores ranging from 65.17 to 98.83 (Aycheh et al., 2023; Facett, 2020), with our sample score falling between this range. Compared to the other subscales, participants rated the following areas of the Emotional Well-being subscale as significantly lower (i.e., barely satisfactory) for their families: (i) stress relief support, (ii) peer group support, and (iii) personal time for one’s interests. Ultimately, SDogs are not an intended intervention for these factors. Although the SDogs reportedly provided benefits to veterans’ families (e.g., comfort), there may be limits to the areas and degree to which they impact family’s quality of life. Further, the reported drawbacks to having a SDog (i.e., stress from managing veterinary bills, food costs, SDog program fees, and having multiple pets in the home), which were similar to past reports (Krause-Parello and Morales, 2018; Nieforth et al., 2021; Yarborough et al., 2018), may have contributed to lower emotional well-being. As the SDogs are further trained and settled in the homes, the family’s emotional well-being may improve.

Caregiving burden results were mixed and contradictory. Past studies have reported CBI mean scores ranging from 40.5 to 56.93 (Su and Chang, 2020; Wasmani et al., 2022), which are relatively lower than the score for the current sample. While CBI scores in the current study provided evidence of caregiving burden and risk of burn-out for participants (Biliunaite et al., 2022), most interviewed spouses reported an overall decrease in their caregiving since the introduction of the SDog in the home. Beyond completing everyday tasks, interviewed spouses also seemed to engage in high levels of monitoring of veterans’ mental states and behaviour. SDogs reportedly helped the veterans gain more independence and decreased the interviewed spouses’ perceived workload, monitoring behaviour, and worrying, but they did not eliminate the caregiver burden for all the spouses, many of whom felt compelled to care for the SDog’s wellbeing and daily needs, too. Spouses’ care for the SDogs may reflect the degree of integration of the dog into the home (Hellings et al., 2022), the realities of SDogs being extra work (Burrows and Adams, 2008), and even the veterans’ decreased mental health. Other researchers have reported similarly mixed results on caregiver burden among veterans’ spouses (e.g., McCall et al., 2020; Nieforth et al., 2021; Nieforth et al., 2022), including Burrows et al. (2016) who concluded that the drawbacks of caregiving for SDogs may be overshadowed by the benefits they bring to spouses of veterans. Training demands, adjusting to life with a SDog, and delayed mental/physical health benefits are other reported drawbacks of SDogs for veterans and caregivers (Yarborough et al., 2018). In their mixed-methods study on veterans’ care partners’ experiences of burden, stress, and support, Rattray et al. (2024) similarly reported divergent and multi-faceted experiences in strain and caregiving burden that shifted over time and were affected by additional burdens such as childcare and finances as well as caregiver age.

### Strengths and limitations

This research was strengthened with the mixed methods approach which enabled data triangulation (Noble and Heale, 2019). This study also advances our understanding of the effects of SDogs on veterans’ families by learning directly from veteran’s family members (Neiforth and Leighton, 2024). However, future observations of SDogs directly in the family home may provide a more fulsome story of family dynamics, integration of the SDog, and even the animal perspective. Measuring a dog’s experiences of SDog-human interactions via techniques such as direct observation, behavioural and body language tracking, or measuring physiological stress indicators (de Winkel et al., 2024; Samet et al., 2022; Völter et al., 2023) could provide valuable insight into the relationship and training needs of veterans and SDogs. Given the SDog training trajectory (i.e., two or more years with continuous maintenance afterwards), longitudinal data collection on the integration of a SDog into a family and its impact on the veteran and family members is pertinent to understanding these dynamics and identifying potential intervention points. Further, half the sample were experienced SDog handlers with unique insights based on their lived/living experiences related to training and working with a SDog over longer periods of time and across a wider range of contexts.

Although the scales were chosen based on content validity and past research, additional scales may provide further insights into the variables of interest. We also did not capture in the current study some additional potentially relevant variables, such as time family members spent with the SDog, SDog training organizations utilized by veterans and their families, and differences in training and support across SDog organizations. We also did not adequately measure caregiver satisfaction, so our findings on this variable are limited. While generalizability was not a goal of our research, it may be limited based on our sample size. However, our findings align with results based on other research samples, so there may be some applicability or transferability (Braun and Clarke, 2021). Time and funding constraints resulted in recruitment difficulties, resulting in a lower than ideal sample size, but neither generalizability nor data saturation were goals as this was an exploratory study. The COVID-19 pandemic may have contributed to the lower sample size as we recruited from a population with potentially minimized capacity to prioritize research participation. It may also have impacted participants’ answers given the volatility of that time.

## Conclusion

SDogs have the potential to not only aid veterans in managing conditions like PTSD but can also offer psycho-social benefits to veterans’ families. Optimizing these individual and familial benefits may require the management of drawbacks of SDogs (e.g., training, caring for their needs, integrating them into families) and recognizing the limits of their role. SDog organizations should consider further development of their programming to account for SDog interactions with veterans’ family. Further research on the integration of SDogs into family homes and their effects on family members is warranted given the varied results of much of the research to date. One future research avenue could be to compare the findings of this study with that of studies in the general companion animal/pet literature for any potential insights. Another research avenue could be to determine whether the current findings matter by severity of PTSD symptoms and/or duration of diagnosis, length of time working with a SDog, and personal views of animals/dogs. If SDogs are intimately integrated into the lives of veterans and their families, there may also be benefit in examining what happens when a SDog passes (e.g., handler and family experiences of grief over the SDog’s death; Bussolari et al., 2024; Gibson et al., 2022; Kogan et al., 2023) and how best to support individuals who experience this loss (DeSantis and Gerlach, 2025).

## Data Availability

The raw data supporting the conclusions of this article will be made available by the authors, without undue reservation.
